# A scoping review: Community health workers engagement in mosquito borne disease prevention activities

**DOI:** 10.1371/journal.pntd.0014621

**Published:** 2026-08-18

**Authors:** Lidia Azurdia Sierra, Emily K. Larson, Adriana Maldonado, Katherine Ellingson, Kacey Ernst, Scott Carvajal

**Affiliations:** 1 The University of Arizona Mel & Enid Zuckerman College of Public Health, Tucson, Arizona, United States of America; 2 Health Promotion Sciences Department, Arizona, United States of America; 3 The University of Arizona Mel & Enid Zuckerman College of Public Health, Tucson, Arizona, United States of America; 4 Epidemiology and Biostatistics Department, Arizona, United States of America; Instituto Pasteur de São Paulo, BRAZIL

## Abstract

**Background:**

Community health workers (CHWs) play increasingly important roles in vector-borne disease prevention globally, yet their contributions remain underutilized and poorly documented in the literature. Understanding how CHWs are deployed across the prevention continuum and identifying barriers to their effectiveness is essential for strengthening mosquito-borne disease control programs.

**Objectives:**

(1) describe the scope and characteristics of studies engaging Community Health Workers (CHWs) in vector-borne disease prevention through community-based strategies; (2) organize the findings by public health prevention levels—primary, secondary, and tertiary—to highlight their impact on CHW knowledge, attitudes, and practices; and (3) identify cross-cutting themes related to facilitators and barriers affecting CHW integration and effectiveness in vector-borne disease control programs.

**Methods:**

This scoping review followed PRISMA-ScR guidelines. Five databases (PubMed, ProQuest, Scopus, Science Direct, and LILACS) were systematically searched for peer-reviewed studies published between 2000 and 2024. Studies were included if they examined CHW-led community-based educational interventions targeting mosquito-borne diseases. Two reviewers independently screened articles using Rayyan.ai software, with an iterative two-stage process: 1. Title, keyword, and abstract review, and 2. Full-text assessment. Data were extracted and organized using public health prevention framework.

**Results:**

Fourteen studies met inclusion criteria. CHWs served critical functions across the disease prevention and control continuum, including health education delivery, early case detection, community mobilization, treatment adherence support, and vector control activities. The evidence synthesis revealed persistent systemic barriers that compromise CHW effectiveness, including insufficient training protocols, resource constraints, and role ambiguity within existing healthcare frameworks. Through an application of a public health prevention frameworks encompassing primary, secondary, and tertiary interventions, this scoping review identified both evidence-based best practices and significant implementation gaps.

**Conclusion:**

The findings demonstrate that while CHWs represent a vital human resource for mosquito-borne disease control, their potential impact remains underutilized due to inadequate institutional support structures. The scarcity of rigorous research reveals a critical gap in the evidence base that must be addressed through systematic evaluation of CHW programs, particularly regarding effectiveness, sustainability, and optimal integration models.

## Author summary

In this scoping review, we examined how community health workers (CHWs) participate in educating communities about mosquito-borne diseases, such as malaria and dengue. Our goal was to understand the types of community-based educational programs that involve CHWs, identify gaps in current research, and explore how future efforts can better support these frontline workers. We analyzed studies published over the last 20 years and found that CHWs are deeply involved in preventing and controlling disease through education, early detection, and community mobilization. They often provide health information to families, support treatment, and help reduce mosquito breeding sites. However, our scoping review also revealed consistent challenges—such as limited training, inadequate resources, and conflicting job duties—that can hinder CHWs’ ability to carry out their work effectively. By organizing the evidence by levels of public health prevention (primary, secondary, and tertiary), we were able to highlight both successful strategies and areas needing improvement. Our findings underscore the importance of investing in CHW training, logistical support, and stronger integration with formal health systems. By addressing these needs, future research and public health programs can enhance the impact of CHWs in reducing mosquito borne diseases, particularly in the most vulnerable communities.

## Introduction

Globally, vector borne diseases account for over 700,000 fatalities every year. Vector borne diseases are caused by parasites, viruses, and bacteria transmitted via vectors such as mosquitoes, ticks, or fleas. In this paper, a vector refers to a mosquito, specifically the *Aedes aegypti*, responsible for transmitting diseases such as chikungunya, dengue, yellow fever, and Zika, and the *Anopheles gambiae* mosquito, which transmits malaria. These deaths could have been avoided through public health prevention programming and community engagement [1]. Communities living in vulnerable situations, such as those with poor housing structures, those without access to healthcare, and those residing in rural areas, are most at risk for contracting and experiencing more severe consequences of disease, as there are often not adequate sanitary vector control measures or infrastructure in place to minimize vector-human contact [2]. Although underserved populations are more at risk for transmission and poor vector borne disease outcomes, there has been insufficient effort from government officials and international organizations to address this health disparity and its causes [3]. Lack of sustained funding, research support, and socio-environmental factors inhibit preventative measures, which leads to increased outbreaks and deepens the burden of vector borne diseases on marginalized communities. Some examples of factors contributing to vector borne disease inequities in marginalized communities include Climate change, globalization, urbanization, insufficient and degrading public health systems, and vector adaptation and resistance to control strategies [3; 4].

Increases in climatic events (i.e., droughts, extreme precipitation) and the profound effects of COVID-19 have only widened the gaps in vector borne disease health disparities, making it increasingly difficult to provide for communities disproportionately affected by such events [5].

Primary strategies to reduce the risk of vector borne disease in humans include vector control, such as larvicide treatment, mosquito traps, education on vector avoidance, and rapid testing and treatment of infections. Barriers to disease surveillance and infection prevention for vector borne disease control can be overcome with novel community-engaged practices to intervene and prevent the spread of vector borne diseases [6]. Community health workers (CHWs) are frontline public health professionals who are trusted members of their communities [7]. The community health worker model consists of a trained workforce of health educators, such as community health workers (CHWs), who receive up-to date information on effective and low-cost community-based prevention strategies to evade diseases carried by mosquitos. They are known by various titles, including community health advisors, lay health advocates, promotoras, outreach educators, community health representatives, peer health promoters, health animators, and peer health educators to name a few [8,9]. Their shared lived experiences with community members position them well to provide various resources, including essential public health services for health risk mitigation and promoting healthy behaviors through campaigns, informal counseling, and advocacy [7,10]. Additionally, CHWs excel at connecting community members with formal healthcare providers and navigating complex healthcare systems [10]. Due to these reasons, CHWs are ideal partners for studies assessing the feasibility and effectiveness of vector borne disease campaigns. Moreover, CHWs live and work in the communities they serve, therefore they are particularly equipped to share knowledge through culturally relevant channels [11].

This scoping review aims to examine how Community Health Workers (CHWs) are engaged in community-based strategies for mosquito-borne disease prevention and control. By synthesizing peer-reviewed literature across primary, secondary, and tertiary public health prevention levels, we highlight the diverse roles CHWs play. In doing so, this study provides a foundation for advancing research and public health strategies that strengthen CHW capacity and improve community-level responses to vector-borne disease threats.

## Methods

Our team constructed this scoping review following the Joanna Briggs Institute (JBI) guidance document on systematic scoping reviews [12,13] and the Preferred

Reporting Items for Scoping Reviews: the five-stage methodological framework by Arksey and O’Malley [14]. Conducting a scoping review to answer this research question was appropriate due to the scant literature on vector borne prevention and education in CHWs globally.

### Search strategy

#### Database Selection and Search Parameters.

We searched five databases for peer-reviewed and gray literature: PubMed, ProQuest, Scopus, Science Direct, and Latin American and Carribean Health Sciences Literature (LILACS). We conducted searches used Medical Subject Headings (MESH) and critical concepts of community-based participatory research (CBPR), mosquito borne disease, and community health workers [8].

### Temporal and linguistic scope

We restricted the study to publications dated from 2000 to 2024. This timeframe was selected to ensure robust data availability and comparability, as publications prior to 2000 were significantly less likely to be digitized and indexed in the databases utilized, which would have introduced substantial selection bias into our analysis.

We limited articles screening to readily available English and Spanish due to the authors fluency in these languages and in recognition of the ethical challenges of appropriately reporting on articles in other languages without personnel expertise to do so. This decision enabled direct engagement with source materials without relying on translation, thereby reducing the risk of misinterpretation or misrepresentation of findings from the included literature.

### Search execution and article management

We collected identified articles in Zotero and conducted blind screening using Rayyan.ai software.

### Screening process

We employed an iterative two-stage screening process:

Stage 1: Title, Keyword, and Abstract Review: We reviewed titles, keywords, and abstracts to determine potential article inclusion based on alignment with study objectives. If there were any disagreements between two of the reviewers a third reviewer would be consulted.

Stage 2: Full-text Review: We conducted a comprehensive review of the full text of each article retained from Stage 1 and extracted population, intervention, comparison, and outcome to generate a literature matrix and further assess its fit with the study objectives and confirm final inclusion. Fig 1 is the PRISMA flowchart which represents the flow of extraction.

### Primary search strategy

The following PubMed search served as the primary search strategy (Table 1). We adjusted for each additional database to match their respective language conventions and keyword requirements:

**Table 1:** Search strategy.

	Description	PubMed search text
Concept 1	Community engagement/ participatory research	“community-based participatory research”[All Fields] OR “community participation”[MeSH Terms] OR “community participation”[All Fields] OR “community-institutional relations”[MeSH Terms] OR “community-institutional relations”[All Fields] OR “cooperative behavior”[MeSH Terms] OR “cooperative behavior”[All Fields] OR “participatory research”[All Fields] OR “participatory action research”[All Fields] OR “community engagement”[All Fields] OR “community engaged”[All Fields] OR “community health partnership”[All Fields] OR “community health partnerships”[All Fields] OR “community academic partnership”[All Fields] OR “community academic partnerships”[All Fields] OR “community partnership”[All Fields] OR “community partnerships”[All Fields] OR “research partnership”[All Fields] OR “research partnerships”[All Fields] OR “community based participatory research”[All Fields] OR “community-based participatory research”[All Fields] OR “community based”[All Fields] OR “community-based”[All Fields]
Concept 2	Community health workers	“community health workers”[MeSH Terms] OR “community health workers”[All Fields] OR “promotora”[All Fields] OR “promotoras”[All Fields] OR “promotores”[All Fields] OR ((“public health”[MeSH Terms] OR “public health”[All Fields] OR (“community”[All Fields] AND “health”[All Fields]) OR “community health”[All Fields]) AND (“outreach”[All Fields] OR “outreaches”[All Fields] OR “outreaching”[All Fields]) AND (“occupational groups”[MeSH Terms] OR “occupational groups”[All Fields] OR “worker”[All Fields] OR “workers”[All Fields]))
Concept 3	Vector-borne diseases/mosquito-borne diseases	“vector borne diseases”[MeSH Terms] OR “vector borne diseases”[All Fields] OR “vector borne disease”[All Fields] OR (“vector”[All Fields] AND “borne”[All Fields] AND “disease”[All Fields]) OR “dengue”[MeSH Terms] OR “dengue”[All Fields] OR “zika virus”[MeSH Terms] OR “zika virus”[All Fields] OR “zika virus infection”[MeSH Terms] OR “zika virus infection”[All Fields] OR “chikungunya fever”[MeSH Terms] OR “chikungunya fever”[All Fields] OR “chikungunya”[All Fields]
Final query	Combine all three concepts	(...Concept 1...) AND (...Concept 2...) AND (...Concept 3...)

## Results

This scoping review identified 14 studies that examined the role of community health workers (CHWs) in mosquito-borne disease prevention and control across diverse geographic settings in Africa, Asia, and Latin America. The studies were published between 2001 and 2021, with sample sizes ranging from small qualitative investigations to large-scale quantitative assessments involving hundreds of CHWs. To systematically analyze the evidence, findings are organized according to the three levels of public health prevention, each representing distinct approaches to disease control and different roles for CHWs. Table 2 showcases 14 studies included in the following analysis.

**Table 2:** Article Summaries.

Citation	Study Aim(s)	Study Design	Nation	Key Finding(s)
[15]	To examine the role of community health workers in mosquito control and surveillance within an urban desert environment in Arizona, USA.	Quantitative	North America	• Surveys conducted before and during the didactic training revealed a noteworthy enhancement in the knowledge of community health workers (CHWs) on diseases carried by mosquitoes and their preventive measures. • Additionally, community health workers successfully collected mosquito larval samples during the first mosquito season, demonstrating the viability of CHW in mosquito monitoring.
[16]	To assess the needs of community health workers that would help sustain and retain their services to enhance the efficient delivery of community care management of malaria (CCMm) in Kenya.	Qualitative	Africa	• Proper incentivizing, refreshing training/updates on malaria, and being equipped with requisite tools (identified in the study) are essential in helping CHWs perform their CCMm tasks better.
[16]	To meet the needs of pregnant women through an IPTp distribution program at the community level by CHWs in Burkina Faso.	Qualitative	Africa	• CHWs reported feeling capable in supporting c-IPTp, opportunity to train and supportive supervision were imperative. • Male and female CHW involvement was beneficial
				• IPTp was feasible and acceptable to HCWs and CHWs and might strengthen the uptake of IPTp and ANC
[17]	To evaluate the performance of Brazilian Community Health Agents when dengue control activities were added to their tasks.	Mixed Methods	South America	• Incorporating the National Dengue Control Program into the established Family Health Strategy is viable and developed without prejudice to dengue control activities but was not consistent across all sites (namely Sao Fabriel do Oeste).
				• This could be due to CHWs additional workload which led to declining performance in the Family Health Strategy activities.
[18]	To assess the knowledge, attitudes, and practices of CHWs involved in malaria prevention and control in Cameroonian communities. Additionally, the study sought to understand the correlates of CHWs’ knowledge regarding malaria prevention and control.	Quantitative	Africa	• The knowledge of community health workers regarding malaria, its causes, and common signs and symptoms is high, but there are still gaps in knowledge regarding specific aspects of malaria prevention. CHWs’ attitudes and practices related to malaria prevention and control are generally positive, but some misconceptions and deficits in practices exist.
				• Only being single had a statistically significant association with having knowledge on malaria prevention and control, highlighting the need for additional education and training for CHWs.
[19]	To determine the diagnostic competence of community health workers in malaria control programs of Nigeria.	Quantitative	Africa	• Malaria was diagnosed by community health technicians with a 0.875% incorrect diagnostic rate (overdiagnosed by CHT). Employers and the populace should place confidence in CHT/W services where they are trained and experienced.
[20]	To explore the knowledge, attitudes, and practices of Community Health Workers (CHWs) about malaria prevention in a selected District of Rwanda. The study aimed to assess the CHWs’ understanding of malaria transmission, symptoms, prevention, and management, with a focus on identifying areas where CHWs may need additional training and support.	Quantitative	Africa	• A moderate level of knowledge about good practices for malaria prevention and management among the CHWs in the selected District of Rwanda. • Study emphasizes the need for improved, accurate knowledge dissemination through appropriate channels to enhance CHWs’ practices in malaria prevention and management. • Additionally, the study highlights the necessity for collaboration with various public and private organizations to ensure effective malaria prevention and management.
[21]	To influence perception health animators in vulnerable populations and encourage self reliance. To describe the process of implementing community workshops on malaria by health animators to improve uptake and use of malaria control interventions in rural Malawi.	Mixed Methods	Africa	• Community workshops may be used as a tool to influence behavior changes to prevent malaria. • Social structures and power dynamics affect community response to health animators. Need identified for systemic monitoring of workshops to ensure implementation fidelity and strengthen health systems.
[22]	To describe the Endemic Disease Control Worker (EDWC) integration process into the Family Health Strategy.	Quantitative	South America	• All participants reported guiding property dwellers and 58 carried out mechanical vector control during the inspection of properties. EDWC integration into Family Health Strategy, 18 participants highlighted teamwork as a positive aspect, and 15 highlighted the lack of autonomy to undertake legal interventions as a negative aspect.
				• EDCW integration in the Family Health Strategy is feasible but adjustments need to be made to optimize activities within the perspective of shared work in the same territorial area.
[23]	To understand a new drug delivery strategy community-directed treatment (comDT) and compare its effectiveness with traditional health services-organized drug delivery	Mixed Methods	Asia	• The distribution and compliance rates were lower under the comDT strategy compared to the traditional health services drug delivery model
[24]	To assess the effectiveness of a community based approach program using community stakeholders in Thailand. Knowledge, perceived susceptibility, self-efficacy, and regular larval survey behavior	Mixed Methods	Asia	• There is strong evidence supporting the effectiveness of a community-based approach program for the prevention and control of dengue hemorrhagic fever (DHF). • The study found that empowering key community stakeholders through active participation in ongoing activities played a crucial role in the success of the program.
				• Additionally, involving community stakeholders in regular monthly meetings, dialogue, and reflection enhanced the program’s outcomes.
[25]	To assess the effectiveness of creating small task forces at the neighborhood level to engage stakeholders in dengue prevention in Cuba. The researchers wanted to evaluate how this approach could lead to specific behavioral and environmental changes and reduce the presence of *Aedes aegypti* mosquitoes. They also aimed to monitor the participation process and its impact on behavioral,environmental, and entomological outcomes.	Quantitative	Caribbean	• Active community participation, facilitated by the creation of local task forces that included all stakeholders, can lead to effective government-community partnerships in dengue prevention. This partnership resolved problems of mutual concern, resulting in decreased household risk behavior, reduction of environmental risks, and effects on entomological indicators. However, the mid-term sustainability of this approach remains to be demonstrated.
[26]	To assess the impact of community-based environmental management, specifically the community working groups (CWG), on Aedes aegypti infestation and dengue fever transmission during the dengue outbreak in Santiago de Cuba in 2006–2007. The study aimed to compare the attack rates of dengue fever in areas where CWGs had been conducting intervention with those where routine vector control activities alone were conducted.	Mixed Methods	Caribbean	• Community-based environmental management, specifically through the implementation of community working groups (CWG), inserted in the routine Aedes aegypti control program, not only sustained vector infestation but also had a significant impact on reducing dengue transmission. • This conclusion was drawn based on the significantly lower attack rates of dengue fever in the intervention blocks compared to the control blocks, indicating a relative risk reduction of 4.5 and a preventable fraction in the control blocks of 78% if the participatory strategy had been implemented there.
[27]	To study the effectiveness of an integrated intervention of health worker training on Aedes spp. mosquito control in Guatemala.	Mixed Methods	Central America	• 88% of the aspiring CHWs were accredited for their increased proficiency with vector control. During the study period, more eggs were captured in ecological ovillantas than in regular ovitraps.
				• Following CHW training, there was an increase in knowledge, enthusiasm, and involvement in community mosquito control and trapping.

### Primary prevention

Primary prevention involves actions taken to protect people who are at risk but not yet affected by a disease. The main goal is to stop the disease from developing in the first place, focusing on individuals who are currently healthy. This approach often includes strategies to reduce exposure to potential hazards or to strengthen the body’s defenses, thereby stopping the onset of illness before any symptoms appear [28]. In the context of vector-borne diseases, this includes community education, environmental management, and measures to prevent mosquito breeding and bites. For example, using insecticide-treated bed nets to prevent mosquito bites is a primary prevention strategy against malaria. By sleeping under these nets, individuals avoid contact with mosquitoes that may carry the malaria parasite, thereby reducing their risk of ever contracting the disease. Two studies [15 and 27] focused on interventions such as mosquito control, education, and community mobilization, often led by community health workers (CHWs).

Arnbrister [15] conducted a comprehensive study in the United States (southern Arizona) examining CHWs’ capacity for *Aedes aegypti* mosquito larval surveillance and disease education. The intervention included intensive training on mosquito biology, disease transmission, and surveillance techniques. While pre-and post-training assessments demonstrated significant improvements in CHW knowledge about mosquito-borne diseases and prevention strategies, the study revealed important implementation challenges. During the initial mosquito season, CHWs successfully collected larval samples and conducted community education activities. However, logistical barriers—such as inability to take the time required when conducting home visits, billing code for service, and competing responsibilities from other health programs substantially reduced their participation in the following season, highlighting the critical importance of sustained organizational support and manageable workload distribution. Building on similar integrated approaches, [27] implemented a multi-component intervention in Guatemala that combined CHW training with innovative mosquito monitoring tools (ovitraps) and structured community engagement activities. The results were promising: 88% of participating CHWs achieved accreditation for improved vector control competency, ecological monitoring showed increased mosquito egg capture compared to standard methods, and community participation in mosquito control activities expanded significantly. [27] demonstrated that when CHWs receive appropriate tools and training, they can effectively bridge the gap between technical mosquito control measures and community behavior change.

The following studies explored more participatory approaches to primary prevention. [21] examined the work of “Health Animators” in rural Malawi, who conducted 2,704 malaria prevention workshops across vulnerable communities. The intervention resulted in measurable behavior changes, increased uptake of preventive treatments, and broader community participation in malaria control activities. Notably, the success of these workshops was significantly influenced by the involvement of respected village leaders, illustrating how social and cultural dynamics can either facilitate or hinder health behavior change initiatives.

In Thailand, Therawiwat et al. [24] employed an action research methodology that emphasized community empowerment through participatory training and ongoing stakeholder engagement.

The intervention led to improvements in knowledge about dengue prevention, increased perceived susceptibility to the disease, and enhanced community participation in larval survey activities. Most importantly, entomological indices dropped significantly in intervention areas, providing concrete evidence of the intervention’s impact on vector populations. The study’s emphasis on continuous community empowerment and regular stakeholder meetings emerged as key factors in sustaining behavior change over time.

The Cuban studies by [26] provide compelling evidence for the effectiveness of structured community partnerships in primary prevention. The initial 2007 study evaluated the creation of neighborhood-level Community Working Groups that brought together diverse stakeholders in dengue prevention efforts. The way in which these groups operated were very closely aligned to community health workers. These groups successfully reduced household risk behaviors, improved environmental risk management, and positively impacted entomological indicators. The participatory model proved particularly effective at resolving community concerns while simultaneously addressing public health objectives.

The 2011 follow-up study provided crucial evidence of long-term impact during a dengue outbreak in Santiago de Cuba. Areas where Community Working Groups had been implementing interventions showed significantly lower dengue attack rates compared to control areas. This demonstrated that well-structured community participation can provide sustained protection against vector borne disease transmission even during epidemic conditions.

Two Brazilian studies highlighted important challenges in integrating vector control activities with existing CHW responsibilities. Cazola et al. (2014) [17] compared two cities with different approaches to incorporating dengue control into Community Health Agents’ duties. While dengue control indicators were maintained in both settings, the city where CHAs managed both dengue control and broader health responsibilities experienced declines in other health services, raising critical questions about optimal workload distribution and task prioritization. Similarly,Pereira et al. (2021) [22] evaluated the integration of Endemic Disease Control Workers into Brazil’s Family Health Strategy. Although the integration improved teamwork and enhanced dengue control activities, participants identified significant legal and coordination barriers that limited their effectiveness. These findings underscore that successful integration requires careful attention to role definitions, legal frameworks, and coordination mechanisms to preserve both disease-specific outcomes and broader public health goals.

Across all primary prevention studies, several key themes emerge. CHW-led interventions show the greatest success when supported by consistent training, adequate logistical resources, and integration with formal health systems. Community engagement and leadership support are essential facilitators; while competing responsibilities and inadequate organizational support represent significant barriers. The evidence suggests that primary prevention interventions require sustained investment and careful attention to implementation context to achieve and maintain their intended impacts.

### Secondary prevention

Secondary prevention focuses on identifying diseases at an early stage, specifically in people who do not yet show symptoms but may already have underlying changes in their bodies [28]. These individuals appear healthy, but tests can detect the disease before symptoms develop. Secondary prevention is commonly carried out through screening programs. For instance, in the context of vector-borne diseases, using rapid diagnostic tests (RDTs) to screen for malaria in communities helps detect infections in people who do not yet feel sick. This allows for early treatment and prevents further spread of the disease. In this scoping review, this typically involves CHWs identifying early symptoms, performing routine testing, and distributing preventive treatment.

Boakye et al. [29] conducted an in-depth qualitative assessment of CHW needs for sustaining community case management of malaria (CCMm) in Kenya. The study revealed that CHWs played central roles in malaria testing using rapid diagnostic tests, treatment distribution, and educational outreach to community members. However, their effectiveness was heavily dependent on reliable access to essential supplies, regular refresher training, appropriate financial compensation, and meaningful integration with formal health systems. The study emphasized that investing in CHW infrastructure—including both material resources and professional development—is essential for maintaining quality secondary prevention services at the community level. Building on similar themes, [16] implemented a community based intermittent preventive treatment in pregnancy (IPTp) program in Burkina Faso. CHWs were trained to identify pregnant women, provide preventive malaria treatment, and link them to antenatal care services. The intervention resulted in improved coverage of preventive treatment and increased access to formal antenatal care. While CHWs generally reported feeling capable of supporting the program, the study reinforced that ongoing training and supportive supervision were critical components. Additionally, the involvement of both male and female CHWs proved beneficial, with female CHWs more effective at engaging pregnant women and male CHWs providing important community credibility and support.

Two studies specifically examined CHW competencies in secondary prevention activities. Habimana et al. (2016; [20]) assessed knowledge, attitudes, and practices regarding malaria among CHWs in Rwanda, finding that most participants demonstrated good understanding of malaria transmission and symptoms. However, significant gaps existed in their prevention and management behaviors, and the study identified important associations between CHW demographics, knowledge levels, and practice quality. These findings highlighted the value of targeted, tailored training interventions that address both knowledge gaps and practice improvement. In Cameroon, [18] surveyed CHWs involved in malaria prevention and control, discovering that while knowledge and attitudes were generally strong, important misconceptions persisted about specific aspects of malaria prevention and management. CHWs appropriately recognized the severity of malaria and supported hospital-based treatment for severe cases, but continued education was needed to correct misunderstandings and reinforce evidence-based practices. The study found that marital status was significantly associated with malaria knowledge, suggesting that personal characteristics and life experiences may influence CHW learning and performance.

The secondary prevention studies collectively demonstrate that CHWs can effectively contribute to early detection and management of vector-borne diseases, but their success depends heavily on systematic support systems. Key requirements include reliable supply chains for diagnostic materials and medications, regular training updates to maintain technical competencies, appropriate supervision and feedback mechanisms, and clear integration pathways with formal health services. The evidence suggests that secondary prevention interventions require more intensive support systems than primary prevention activities due to their technical complexity and the critical importance of accurate diagnosis and treatment decisions.

### Tertiary prevention

Tertiary prevention focuses on managing disease after symptoms have appeared, aiming to lessen its severity and prevent complications. Unlike secondary prevention, which tries to stop the disease before it fully develops, tertiary prevention works to minimize the long-term impact once the illness is already present. This often involves rehabilitation and ongoing care to improve quality of life [28]. For example, dengue fever, tertiary prevention might include supportive treatments and physical therapy for patients who develop severe complications such as dengue hemorrhagic fever or prolonged joint pain, helping them recover and reduce lasting disability.

[23] conducted a comparative study of drug distribution strategies for lymphatic filariasis control in Tamil Nadu, India. The research compared community-directed treatment (ComDT), where community members similarly described as community health workers took primary responsibility for medication distribution, with traditional health service-organized treatment (HST), where formal health workers-maintained control over the distribution process. Both approaches achieved similar coverage rates (68% for ComDT versus 74% for HST), indicating that community-based distribution could reach comparable proportions of the target population. However, drug consumption compliance was slightly higher in the health service-led approach (59% versus 53%), suggesting that formal health system involvement may improve treatment adherence.

The study’s findings have important implications for tertiary prevention strategies. While community participation proved vital for achieving broad population coverage, the slightly superior compliance rates in the health service-led approach suggested that a hybrid model combining community engagement with centralized coordination might optimize both reach and treatment effectiveness. The authors concluded that health service-led approaches with strong community engagement components may be more efficient and scalable for large-scale treatment programs, while still maintaining the community trust and participation that are essential for program success.

### Synthesis of data

**Fig 1:**
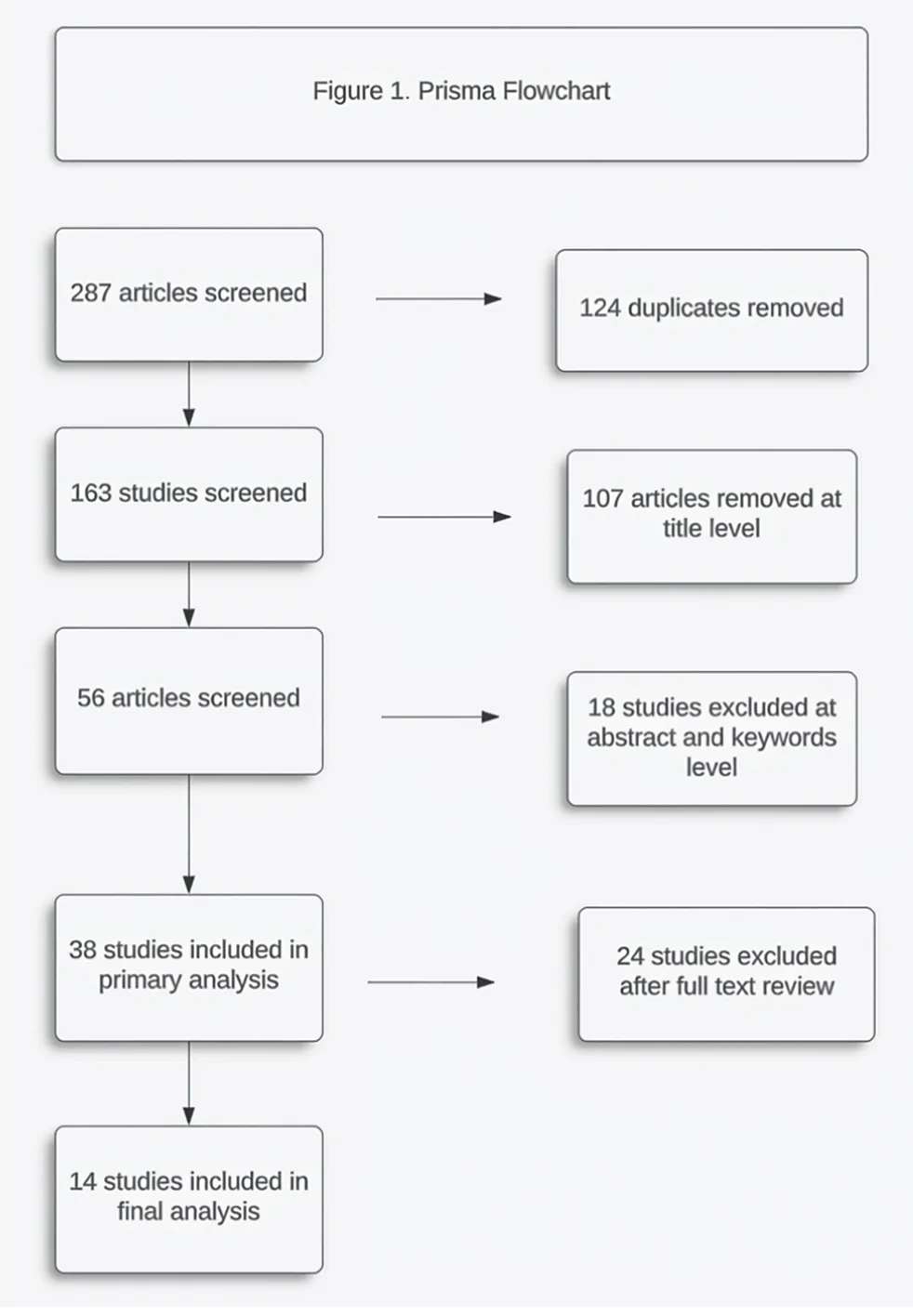
PRISMA flowchart.

## Discussion

This scoping review of 14 studies across Africa, Asia, and Latin America reveals the crucial role of community engagement—particularly through community health workers (CHWs)—in addressing vector-borne diseases. The evidence spans the spectrum of public health strategies, from primary prevention to tertiary care, and highlights both the challenges and facilitators of effective intervention.

Primary prevention efforts frequently rely on CHWs and community leaders to promote behavioral change, reduce vector habitats, and disseminate health information. These strategies are most successful when CHWs receive consistent training, logistical support, and are integrated into broader health systems. For example, studies by [15] and Oliveira Cazola et al. (2014) demonstrate that while CHWs are instrumental in mosquito-borne disease prevention, their effectiveness can be compromised when outbreaks divert attention from other essential services, such as reproductive health education. This underscores the need for comprehensive and ongoing training.

Secondary prevention centers on early detection and case management by CHWs. Sustained impact in these areas depends on continuous education, supportive supervision, and alignment with formal health services. [18] found that knowledge gaps among CHWs—such as misconceptions about malaria transmission—can undermine prevention and management efforts, further emphasizing the importance of robust training and supervision.

Tertiary prevention strategies, such as drug distribution programs, benefit from a hybrid approach that combines community involvement with top-down coordination. This ensures better treatment compliance and operational feasibility, as highlighted in several studies.

Despite their central role, CHWs face significant barriers. Operational challenges, such as inadequate training and poor engagement with community leaders, can limit program success [23; Oliveira Cazola et al., 2014]. Social factors—including reluctance to accept medications and community group conflicts—further complicate interventions. Gender dynamics also play a role; [16] found that while female CHWs are more effective with pregnant women, male CHWs often have greater community influence, potentially leading to uneven outcomes.

Conversely, several facilitators enhance the effectiveness of CHW-led interventions. Strong community involvement, regular collaboration between CHWs and healthcare workers, and a reliable supply of medications are key [16]. Training and empowerment— through fair remuneration, supportive tools, and ongoing education—are also vital [21,29]. Community workshops and local leadership can drive positive health behaviors and sustain malaria control efforts.

Policy-level support, such as the establishment of local task forces and government community partnerships, further strengthens interventions. For instance, Toledo et al. (2007;  [25]) and Habimana et al. (2016) highlight the value of diverse stakeholder engagement and targeted educational initiatives in improving vector control and child health outcomes.

Across all levels of prevention, the centrality of community participation—particularly through well-supported CHWs—is clear. However, for these interventions to succeed, they must be structured, adequately resourced, and continually adapted to local contexts. Addressing operational, social, and knowledge barriers while leveraging facilitators such as training, community engagement, and policy support will be critical. Together, these studies build a compelling case for expanding and optimizing CHW-led strategies in the fight against vector borne diseases.

### Limitations and future research

Of note, the state of the literature on community health workers’ role in vector borne disease control is not yet sufficient to evaluate their overall role or effects across studies and contexts. Thus, we conducted a scoping review, to assess the state of the evidence, and clarify barriers and facilitators to their use in prevention and control programs. While scoping reviews are valuable for mapping the existing literature and identifying research gaps, they have inherent limitations. One notable limitation is the potential for selection bias in including studies that were solely academic research and published within specified databases. However, we tried to mitigate this through software use (e.g., multiple search engines) and other methods recommended [26]. Nonetheless, the review process may inadvertently omit relevant research due to restrictive search criteria or exclude non-English and non-Spanish language studies. Additionally, variability in study quality and methodological rigor across included articles can impact the reliability and generalizability of the findings and make synthesis across divergent methods and contexts. Scoping reviews tend to be appropriate for literature where a detailed assessment of study outcomes may not be informative, focusing instead on broad trends and themes, which may limit the depth of insights into specific intervention effects.

Future research should prioritize several areas. First, large-scale implementation studies are needed to understand how successful community-based interventions can be scaled up while maintaining effectiveness. Second, more research is needed on tertiary prevention approaches and long-term disease management roles for CHWs. Additionally, exploring intersectoral collaboration between community health workers and community animal health workers using One Health frameworks could strengthen approaches to zoonotic vector-borne disease control. Third, comparative studies examining different models of CHW integration with formal health systems could inform optimal approaches to program design. Finally, economic evaluations of different CHW program models would provide important evidence for resource allocation decisions.

## Conclusions

This scoping review achieved its three objectives by: (1) characterizing 14 studies across diverse geographic and methodological contexts, (2) systematically organizing evidence by public health prevention levels to highlight distinct CHW roles and requirements, and (3) identifying persistent challenges in training sustainability, resource adequacy, and health system integration alongside facilitators including community engagement and supportive supervision that must inform future research and program development.

Notably, the identification of only 14 studies meeting our inclusion criteria across reveals a significant gap in the peer-review literature. This scarcity of rigorous research is particularly concerning given the global burden of vector-borne diseases and the widespread deployment of CHW programs in endemic regions. The limited evidence base underscores an urgent need for more systematic research to evaluate CHW effectiveness, optimal implementation strategies, and long-term sustainability of programs in vector-borne disease contexts. Although the evidence base is limited, several studies reported similar challenges related to training sustainability, resource adequacy, and health system integration across different settings, suggesting recurring implementation barriers that merit further investigation for future research and program planning.

Furthermore, this scoping review demonstrates that CHWs can play important roles across all levels of vector-borne disease prevention, but with varying requirements for training, supervision, and support systems. The included studies indicate that CHW’s involvement in vector control programs, particularly in primary prevention activities that can be implemented with relatively limited technical support. However, successful programs require substantial investment in community engagement, systematic training, and integration with formal health systems. As global efforts to control vector-borne diseases continue to evolve, CHW programs represent a critical component of comprehensive control strategies, but only when implemented with appropriate attention to support system requirements and integration challenges.

Future research should prioritize several areas to address the identified gaps in evidence. First, large-scale implementation studies are needed to understand how successful community-based interventions can be scaled while maintaining effectiveness. Second, more research is needed on tertiary prevention approaches and long-term disease management roles for CHWs. Third, exploring intersectoral collaboration between community health workers and community animal health workers using One Health frameworks could strengthen approaches to zoonotic and vector-borne diseases control, including integration into surveillance-response systems that bridge human and animal health sectors. Finally, comparative studies examining different models of CHW integration with formal health systems could inform optimal approaches to program design, and economic evaluations of different CHW program models would provide important evidence for resource allocation decisions.

## Supporting information

S1 Prisma checklist From: Tricco AC, Lillie E, Zarin W, O’Brien KK, Colquhoun H, Levac D, et al. PRISMA Extension for Scoping Reviews (PRISMAScR): Checklist and Explanation. Ann Intern Med. 2018;169:467–473. doi: 10.7326/M18-0850.
(DOCX)
